# Adapting the engine to the fuel: mutator populations can reduce the mutational load by reorganizing their genome structure

**DOI:** 10.1186/s12862-019-1507-z

**Published:** 2019-10-18

**Authors:** Jacob Pieter Rutten, Paulien Hogeweg, Guillaume Beslon

**Affiliations:** 1 Theoretical Biology and Bioinformatics group,Utrecht University, Padualaan 8, Utrecht, Netherlands; 2 Université de Lyon, INRIA, CNRS, INSA-Lyon, Beagle Team, LIRIS, UMR5205, Villeurbanne, 69601 France

**Keywords:** Bacteria, Mutators, Robustness, Genome structure, in silico Experimental evolution

## Abstract

**Background:**

Mutators are common in bacterial populations, both in natural isolates and in the lab. The fate of these lineages, which mutation rate is increased up to 100 ×, has long been studied using population genetics models, showing that they can spread in a population following an environmental change. However in stable conditions, they suffer from the increased mutational load, hence being overcome by non-mutators. However, these results don’t take into account the fact that an elevated mutation rate can impact the genetic structure, hence changing the sensitivity of the population to mutations. Here we used Aevol, an in silico experimental evolution platform in which genomic structures are free to evolve, in order to study the fate of mutator populations evolving for a long time in constant conditions.

**Results:**

Starting from wild-types that were pre-evolved for 300,000 generations, we let 100 mutator populations (point mutation rate ×100) evolve for 100,000 further generations in constant conditions. As expected all populations initially undergo a fitness loss. However, after that the mutator populations started to recover. Most populations ultimately recovered their ancestors fitness, and a significant fraction became even fitter than the non-mutator control clones that evolved in parallel. By analyzing the genomes of the mutators, we show that the fitness recovery is due to two mechanisms: i. an increase in robustness through compaction of the coding part of the mutator genomes, ii. an increase of the selection coefficient that decreases the mean-fitness of the population. Strikingly the latter is due to the accumulation of non-coding sequences in the mutators genomes.

**Conclusion:**

Our results show that the mutational burden that is classically thought to be associated with mutator phenotype is escapable. On the long run mutators adapted their genomes and reshaped the distribution of mutation effects. Therewith the lineage is able to recover fitness even though the population still suffers the elevated mutation rate. Overall these results change our view of mutator dynamics: by being able to reduce the deleterious effect of the elevated mutation rate, mutator populations may be able to last for a very long time; A situation commonly observed in nature.

## Background

Variation and selection together form the core engine of evolution. However, their contributions to the engine are different. While variation fuels the engine with raw energy, selection regulates it and transform this raw energy into the “evolutionary kinetics”, generally – but not systematically – forward, i.e., toward fitness improvement. Consequently, regulating the evolutionary kinetics requires to play on both sides: regulation of variability and regulation of selection. Ultimately, heritable variations are caused by mutations altering the genetic sequence. This process is indeed regulated: it is well known that all organisms reduce their mutation rates by proofreading and repair mechanisms. On the opposite, mechanisms that temporarily or permanently increase the mutation rates have been described in many species and especially in bacteria [1, 2]. In many situations, including experimental evolution [3] and clinical isolates [4], lineages have been observed that present alterations of their repair pathways, resulting in 50 × to 100 × increase of their mutation rates. Similar elevated mutation rates are also observed in endosymbiotic bacteria [5] where they have been suggested to trigger genome streamlining [6].

Increasing the mutation rate by several orders of magnitude is a high risk strategy for a bacteria: It increases the probability to find beneficial mutations at the price of detrimental accumulation of deleterious ones. This risk is classically associated with how well bacteria are adapted to their current environment. When they are poorly adapted, bacteria can quickly evolve a raised mutation rate known as the stress response [7], hence acquiring the “mutator” phenotype. Indeed, mutator phenotype has been suggested to be directly selected for under poor conditions [8]. Additionally, the mutator phenotype appears in novel environments ranging from the presence of antibiotics [9] to the immune response of the infected host [10] or the acquisition of novel functions (such as the incorporation of citrate into metabolism [11]). On the opposite, when well adapted, mutators have been shown to evolve to lower mutation rate. This prevents deleterious mutations from reversing the gained fitness [12].

In modeling terms, novel environments are thought to move the population away from their previous fitness peak in the fitness landscape. In strictly asexual organisms without horizontal gene transfer, when there are many beneficial mutations, the speed of evolution is limited by the time between the discovery of new beneficial mutations in a single lineage. Mutator lineages can accumulate mutations more rapidly than their non-mutator counterparts and outcompete them [3, 13]. Indeed, modeling has shown that mutator identity can be expected to hitchhike with improvements, provided enough possible improvements still exist and the population is large enough [14]. However, when beneficial mutations become too rare (i.e. when the population gets closer to the optimum), the high number of mutations in the mutators offspring will prove deleterious [15] and the subsequent decrease of the effective population size will limit the fixation of favourable mutations [16]. If a well adapted population cannot filter out deleterious mutations, they’ll accumulate and bring the population back to the mutation-selection balance: Mutators would be expected to bring an increased mutational load, hence being counter-selected on the long run in a constant environment.

Rich Lenski’s Long Term Evolutionary Experiment (LTEE) provides a test to the theory. In this experiment 12 strains of *E. coli* evolved independently in a glucose-minimal medium for 30 years. Mutator phenotypes with a 50-100 fold increase of mutation rates have been observed to appear in 6 out of the 12 parallel runs [17]: Mutator identity have hitchhiked along with the improvements in fitness as the bacteria adapted to the new conditions [18].

However, counter to classical expectations, in the LTEE some mutator populations have been shown to take over relatively late in evolution. In some of the repetitions, they have been observed to appear over 20,000 generations after first being exposed to the new (stable) environment, when the increase in fitness has already started slowing down [17]. Additionally, once established a raised mutation rate can stay in the population for long periods of time under the isolated conditions the small populations are kept in, even though compensatory mutations can reduce the mutational rate [12]. Moreover, mutator phenotypes are observed ubiquitously and often at high frequencies in natural environments [1]. These observations raise the question of the fate of mutator phenotypes once established in stable environments: how do well adapted populations that have previously adopted the mutator identity continue to evolve and cope with the increased mutational load? Indeed, all models predicting their conditions of fixation [14, 19, 20] do not take into account possible adaptation to the mutator identity through e.g., reorganization of their genotype. On the opposite, several studies have shown that the mutational robustness of an organism is directly linked to its genomic structure [21, 22].

To tackle this question we have used the Aevol in silico Experimental Evolution platform [22–24] in order to study the fate of a population of bacteria acquiring the mutator identity while already close to a stable optimum, hence mimicking the situation observed in the LTEE Ara-1 population where the mutator phenotype has been acquired after generation 25,000 [12, 17]. By simulating the long-term fate of mutators, we study how the populations respond to high mutation rates by reorganizing their genome and therewith their mutational neighborhood on the fitness landscape.

## Results

### Evolution of the Wild-Type strains

Starting from naive individuals at Generation 0 (see “Methods”), all WT strains quickly adapt, getting close to the phenotypic target in less than 100,000 generations (Additional file 1: Figure S1). As already shown in [23], given the mutation rates used in our experiments, the genomes converge toward bacteria-like structures (see Table 1), although the genomes remain small compared to real bacterial ones: genomes contain a “large” number of genes (mean: 95 genes) organized in operons (with a mean of 1.83 genes per coding RNA) and typically 80% of the genome is coding. This genomic organization is similar to *E. coli* [25]. At generation 300,000, all populations are well adapted to their environment even though there is still room for further adaptation (other experiments with the model show that gradual improvement can continue for more than 10,000,000 generations in a constant environment – data not shown). Indeed, the speed of adaptation substantially slows-down after the first 100,000 generations (Additional file 1: Figure S1). We thus consider that all wildtypes have been sufficiently pre-evolved at generation 300,000 to start the in silico experimental evolution.

**Table 1:** properties of the best individual of each WT strain at generation 300,000

WT ID	Metabolic error	Genome length	RNAs	Genes	Coding fraction
1	0.0079	6517	54	78	0.84
2	0.011	7222	65	80	0.82
3	0.0068	7494	48	95	0.81
4	0.0072	7340	50	82	0.85
5	0.0057	8957	45	105	0.77
6	0.0070	8053	47	101	0.80
7	0.010	7457	46	88	0.75
8	0.0098	10539	63	140	0.82
9	0.0071	8914	53	101	0.83
10	0.0099	8022	50	83	0.82
Mean	0.0082	8052	52.1	95	0.811

### Evolution of the mutator strains

**Fig. 1:**
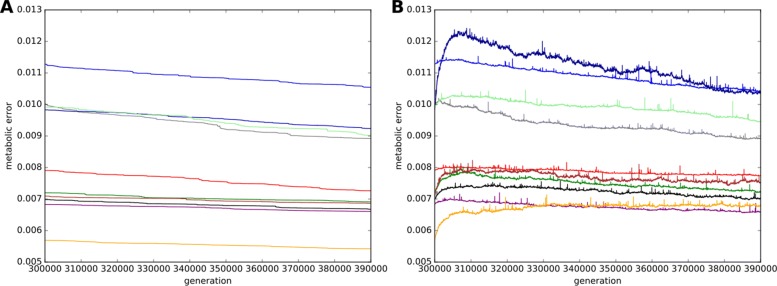
Evolution of the metabolic error in the control and mutator experiments. Mean change in metabolic error of the ancestral lineages between generations 300,000 and 390,000 for the 20 replicates of each WT (see Additional file 1: Figure S6 for the metabolic error of individual replicates). **a** Control strains (10 replicates). **b** Mutators strains (10 replicates). **Color code:** Red: WT1; Light blue: WT2; Purple: WT3; Dark green: WT4; Orange: WT5; Black: WT6; Grey: WT7; Dark blue: WT8; Brown: WT9; Light green: WT10

All WT strains have been cloned 20 times and propagated for 100,000 further generations in the same constant environment. Among these 20 populations, 10 were carrying the mutator phenotype (point mutation rate increased 100 × - see “Methods”) while the remaining 10 were control experiments (keeping the same mutation rate as the WT strains). Figure 1 shows the evolution of the metabolic error for the 100 mutators and the 100 control ancestral lineages averaged for each Wild-Type (ancestral lineages were reconstructed from the fittest individual at the end of the each experiment – see “Methods”). Since the populations coalesce within less than 10,000 generations, the ancestors between generations 300,000 and 390,000 are used as the representative throughout the results from now on, allowing us to study the short and long term effects of mutations in a direct line of descent. As explained above, the control populations are already well adapted. They can still slightly improve (Fig. 1a), but at a rate that is considerably lower than the initial improvement rate of the wild-types. In contrast, the mutator populations experience a rapid increase of the metabolic error due to the 100 fold increase of the point mutation rate (Fig. 1b). However, surprisingly, this effect does not last for the whole experiment: after a while (generally a few thousands of generations, see Additional file 1: Figure S2), the metabolic error of the mutators decreases again, often (62/100) reaching at generation 390,000 values below the ones of their non-mutator ancestor at the beginning of the experiment (generation 300,000).

**Fig. 2:**
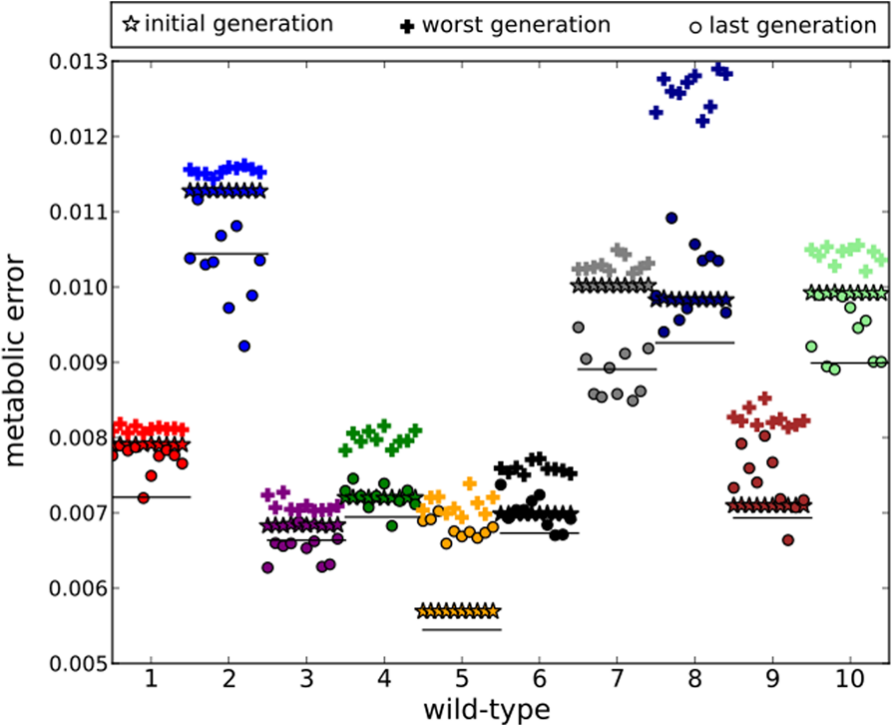
Extreme values of the metabolic error during the 100,000 propagation experiments of the mutator clones. **Stars**: Metabolic error of the ancestor at generation 300,000. **Plus signs**: Worst metabolic error reached in the mutator ancestral lineages. **Circles**: Metabolic error of the mutators at generation 390,000. **Solid horizontal lines**: Mean metabolic error of the control clones at generation 390,000. All mutator clones experience a strong increase of their metabolic error which intensity is clearly WT-dependent. However, among the 100 mutator lineages, 62 completely recovered, showing a lower metabolic error at generation 390,000 than at generation 300,000. From those, 25 improved beyond the mean improvement of the control lineages at generation 390,000

Figure 2 shows the metabolic error of the ancestor at generation 300,000 (stars), the worst metabolic error in the lineage (+ signs, we took the median per 100 generations to exclude extremely negative mutations that are quickly compensated), and the final metabolic error at generation 390,000 for the 10 mutator replicates of each WT population (circles). It shows that all mutator clones experienced the mutational burden (the increase of the metabolic error being mostly WT-dependent, yet not dependent on the WT metabolic error at generation 300,000) and that a partial recovery of the metabolic error occurred in all mutator populations: At generation 390,000, 62 out of the 100 replicates have reduced their error beyond the initial metabolic error of the replicate at generation 300,000, and 25 of those have reduced the error beyond the average metabolic error reached at generation 390,000 by the 10 control replicates originating from the same population (solid horizontal line). A notable exception is the fittest wild-type (WT5) for which none of the mutator strains fully recover the ancestral metabolic error (note that these strains are also the ones for which the burden takes the longest time to reverse – see Additional file 1: Figure S2 leaving it an open question if it would be possible for these mutator stains to completely recover if given enough time).

### Evolution of the genomic structures in the mutator strains

The evolutionary dynamics of the mutators show that, although the mutator identity is always harmful at short term (in a constant environment), it can be neutral or even beneficial with respect to the ancestral fitness on the long run. This even holds in populations originally well adapted to their environment and raises an important question: How did the mutator clones cancel the deleterious effect of the elevated mutation rate and recover their initial fitness. Since it has been shown that the mutational robustness of an organism can be directly dependent on its genomic architecture [22], we first looked at the evolution of genome length, coding length and non-coding length during the 90,000 generations of the experiment.

**Fig. 3:**
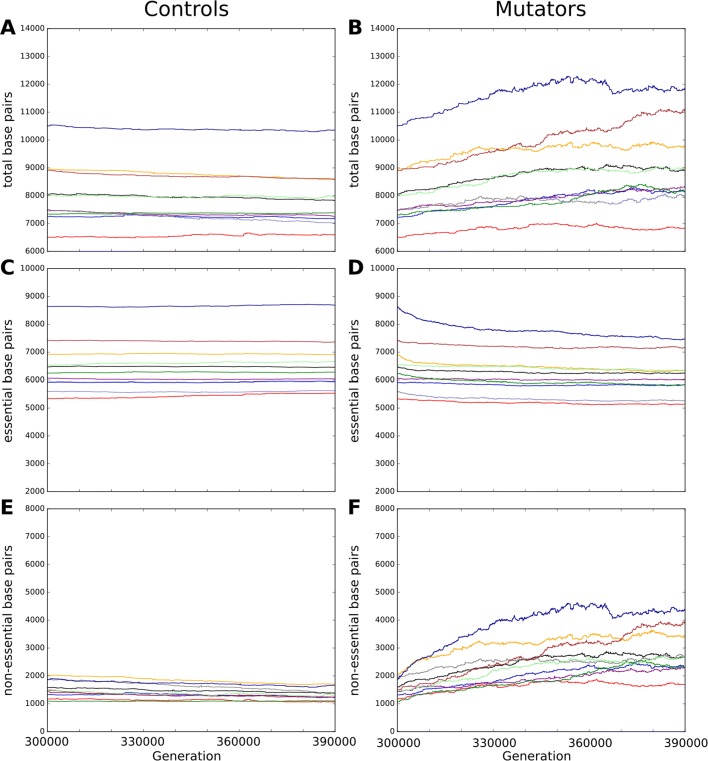
Evolution of genome structure in the ancestral lineages. Evolution of the genome structure in the ancestral lineages of control and mutator populations between generation 300,000 and 390,000. **a** Genome size in the control populations. **b** Genome size in the mutator populations. **c** Number of essential base-pairs in the control populations. **d** Number of essential base-pairs in the mutator populations. **e** Number of non-essential base-pairs in the control populations. **f** Number of non-essential base-pairs in the mutator populations

As Fig. 3 shows, the evolution of genome length and structure strongly differs between the mutator strains and the control strains. While we observed no general trend across the average variation of the full genome size in the controls lineages (Fig. 3a), all mutators strains showed an increase of the genome length with a mean increase of 954 bp (Fig. 3b and Additional file 1: Table S2) resulting in a significantly larger genome (Wilcoxon signed rank test, *p*-value <0.002). This contradicts intuition, which suggests controls would increase their genome size as they became fitter, while mutators would lose parts of their genome that they were unable to retain, hence losing fitness. However, both these predicted effects concern the coding sequence. We thus had a closer look at the evolution of the length of the two main genomic compartments: essential “coding” genome and non-essential “non-coding” genome (see “Methods”).

In accordance with the initial intuition, the number of essential base pairs slightly increase in the control clones (mean increase: 31 bp, Fig. 3c and Additional file 1: Table S2) while it clearly decreases in the mutator clones (mean decrease: 359 bp, Fig. 3d and Additional file 1: Table S2) resulting in a significantly smaller essential genome (Wilcoxon signed rank test, *p*-value <0.002). Interestingly, in the mutator clones the number of essential base pairs declined all along the experiment even when the clones started to recover. This is accomplished both through a reduction of the number of transcribed sequences and a reduction of genetic material (both in number of genes and in base pairs per gene, see Additional file 1: Table S3). This demonstrates the extent to which these populations could optimize their coding structure under the mutational pressure and encode more information in a shorter sequence.

Looking at non-essential base pairs, control and mutator clones also behave completely differently. The control clones consistently reduced their number of non-essential base pairs (mean decrease of 203 bp - see Fig. 3e and Additional file 1: Table S2) but strikingly the mutator clones strongly increased the length of the non-essential compartment (mean increase: 1314 bp, see Fig. 3f and Additional file 1: Table S2). This results in a significantly larger non-essential genome in the mutators (Wilcoxon signed rank test, *p*-value <0.002) which is the source of the net increase of their genome size.

Per definition, non-essential base pairs do not contribute to the phenotype and do not affect the metabolic error directly (see “Methods”). In a first approximation, mutations affecting non-essential base pairs are neutral. However, the size and frequency of chromosomal rearrangements are coupled to genome size, including essential and non-essentials base pairs. Even though an increase of the number of non-essential base pairs is neutral, it induces an effect on rearrangements that are, in mean, longer and more numerous, hence increasing the genome variability and decreasing genome robustness [22, 26]. Hence, we observe a paradoxical response to the 100 × increase of point mutation rate. In line with expectations, we observe an increase of robustness to point mutations (through streamlining of the essential genome). However mutators evolve simultaneously a decrease of robustness to chromosomal rearrangements that are, in mean, larger and more numerous due to the accumulation of non-essential base pairs.

### Analysis of the fixed mutations

**Fig. 4:**
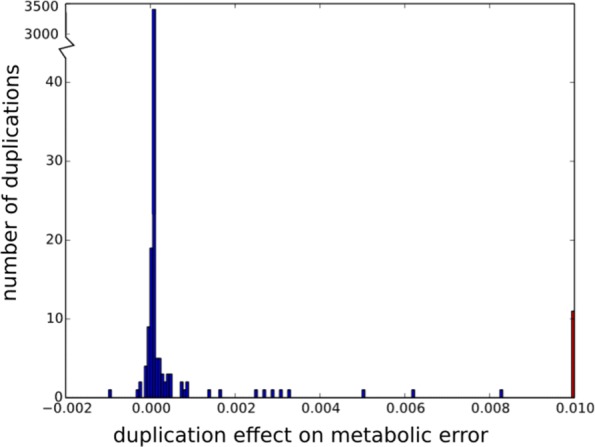
Distribution of the effect of duplications on metabolic error in the ancestral lineages of all the mutator populations. Duplications are deleterious when they increase metabolic error (i.e., variations are positive). The large red bar of error rate increasing events at the right edge shows the prevalence of deleterious duplications with effects equal to or larger than 0.01

The simplest hypothesis to explain the increase of non-essential genome is that these sequences accumulate through an hitch-hiking process: if the mutator clones undergo many favorable events that increase genome size (e.g. gene duplication), non-coding sequences may accumulate without having any direct or indirect effect on the fitness. In Aevol, there are two kinds of mutational events that can contribute to an increase of the genome size: small insertions and large duplications. Looking at the number and size of the fixed events, it clearly appears that, although small insertions are more numerous than large duplications (Additional file 1: Table S4), the former only marginally contribute to the variation of genome size increase while the latter clearly drive the genome inflation (Additional file 1: Figure S3). We thus measured the effects of large scale duplications on fitness in the ancestral lineage of the mutator clones. Figure 4 shows that there is no tendency for duplications to reduce the metabolic error. Globally, during the 90,000 generations of the experiment, the 100 mutator clones together fixed 4140 large duplications (Additional file 1: Table S4), the overwhelming majority of which being exactly neutral (Fig. 4). Only a tiny fraction of the duplications have a positive effect (i.e. reduce the metabolic error) and a much larger proportion is deleterious, with often strongly deleterious effects (right bar on Fig. 4). Therefore, increase of the genome size cannot be due to non-essential base pairs hitch-hiking favorable duplications. On the opposite, the growth of the non-essential part of the genome is in spite of, rather than driven by, the direct effect of duplications on fitness.

With direct fitness effects excluded, we turned our attention towards the indirect effects. To study this we focused on how non-essential base pairs affect the fitness landscape.

**Fig. 5:**
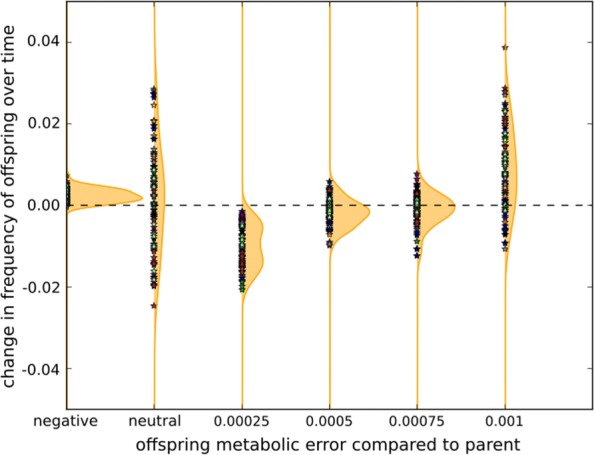
Difference in mutational neighborhood of the mutator clones between generation 390,000 and 300,000. For each mutator lineage 2 million offspring were generated from the ancestor at generation 300,000 and 2 million from the ancestor at generation 390,000 (see “Methods”). For each offspring, we measured *Δg*, the variation of metabolic error relatively to its parent. We then binned these differences in 6 bins and measured the variation of frequency in these bins between generations 300,000 and 390,000 for each lineage (Stars). Areas represent the maximum density of presence in the plot. We observe a slight increase in the number of offspring that gain fitness relatively to their parent (negative variation of metabolic error), no variation of neutral offspring, a decrease in slightly deleterious offspring (bin 0.00025) and an increase in highly deleterious offspring (bin 0.001)

It is well known that the fate of an evolving population depends on the relationship between the rate of mutation accumulation and the local shape of the fitness landscape [21]. Moreover, the variations of the genomic structure is likely to change this local shape by e.g. increasing the proportion of neutral or deleterious events [22, 26]. To measure this effect, we sampled the mutational neighborhood of the mutator clones at generations 300,000 and 390,000 (see “Methods” section). We then compared the frequency of offspring with a larger/same/lower fitness (i.e. lower/same/larger metabolic error respectively) than their parent at both time points: Fig. 5 presents the distribution of the variation of the differences between parents and offspring metabolic error between generations 300,000 and 390,000, binned in 6 bins. First, the fraction of offspring showing an increased fitness (i.e. a negative variation of the metabolic error) comparatively to their parent is slightly increased during the experiment (bin “negative”). However, since the Wild-Types are well adapted to their environment, it remains very low (0.31% at generation 300,000 and 0.55% at generation 390,000), hence it is strongly dependent on sampling errors and the variation is difficult to analyze. Similarly, there is no variation of the neutral fraction (bin “neutral”) that remains close to 37% in mean during the experiment. However, surprisingly, we observe a net decrease of the fraction of offspring undergoing a small loss of fitness (bin “0.00025”) and a net increase of the fraction of offspring undergoing a strong loss of fitness relatively to their parents (bin “0.001”).

### Effect of non-essential genome in evolution on robustness and anti-robustness

**Fig. 6:**
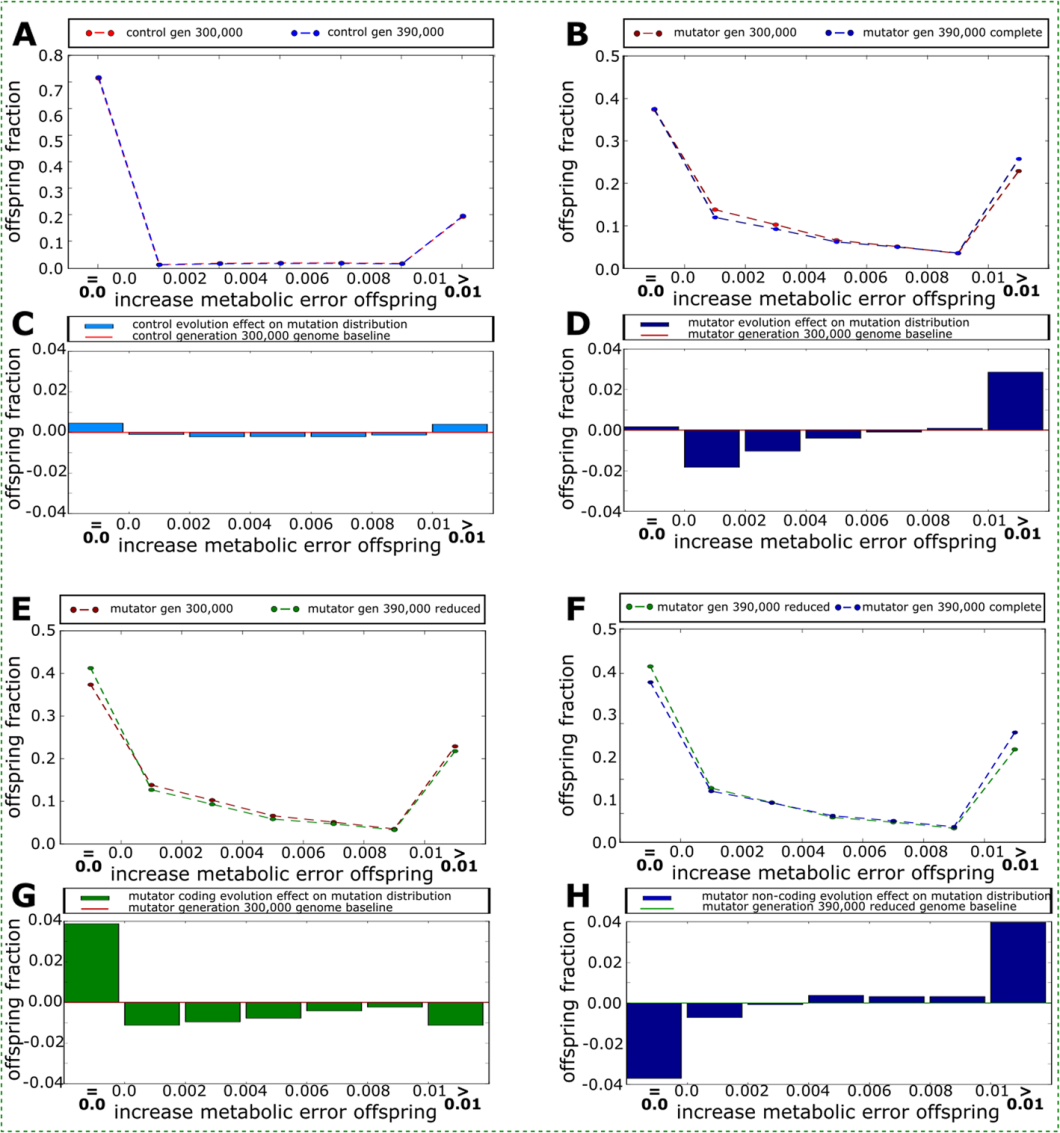
Top: Evolution of the mutational neighborhood of the Control (**a**, **c**) and the Mutator (**b**, **d**) lineages between generations 300,000 (red) and 390,000 (blue). In both cases the distribution of offspring evolves a deeper “U-shape” where the vast majority of offspring are either neutral or highly deleterious. In the Control lineages the increase in neutral offspring and highly deleterious offspring is balanced. In the Mutator strains, the U-shape becomes right skewed towards highly deleterious mutations and there is a strong reduction of slightly deleterious offspring. Bottom: Evolution of the mutational neighborhood of artificially reduced Mutator genomes (at generation 390,000) compared to the Mutator lineage at generation 300,000 (**e**, **g**) and 390,000 (**f**, **h**). The former comparison enables to isolate the effect of coding genome streamlining (**g**). The latter comparison enables to isolate the effect of non-coding genome expansion (**h**). The coding genome evolves to be far more neutral, skewing the distribution to the left (**g**). Non-coding genome expansion cancels most of the former effect and strongly skews the distribution to the right (**h**). These two effects (**g**, **h**) sum up to the right skew observed in (**d**). All distributions are binned in 7 bins. The first bin contains neutral offspring (*Δg*=0). Bins 2 through 6 contain the intermediately deleterious offspring binned in 0.002 intervals (*Δg* from 0.002 to 0.004; 0.004 to 0.006; 0.006 to 0.008 and 0.008 to 0.01). The last bin contains all heavily deleterious offspring (*Δg*>0.01). See main text and Methods for details

Figure 5 showed two opposite effects of mutator identity on the distribution of offspring fitness: An increase in highly deleterious offspring and a decrease in slightly deleterious offspring. To contrast this evolution with that of the controls, we plotted the distribution of deleterious offspring at generations 300,000 and 390,000, binned in 7 bins, for the different experiments (Fig. 6). This shows how the mutational neighborhood of the populations have varied in the different conditions. The mutational neighborhood of the controls is shown in Fig. 6a. Both at generation 300,000 and 390,000, the majority of offspring either conserve the fitness of their parent or have undergone highly deleterious mutations, resulting in a “U-shape” distribution. This is a common motif when the evolving genome structure can itself evolve [27]. In the control lineages, the variations of the “U-shape” between generation 300,000 and 390,000 is a symmetric deepening: Neutrality increases as well as the number of highly deleterious offspring (hence depleting the number of intermediately deleterious offspring – Fig. 6c). When looking at the mutators, the “U-shape” is not simply becoming deeper during the 90,000 generations of the experiment: It evolves a right-skewed distribution: The number of neutral offspring increases only minimally while the number of slightly deleterious mutations decreases and the number of highly deleterious mutations increases (see Fig. 6b, d). However, this dynamic is specific of mutator strains that were pre-evolved under low mutation rates. Indeed, in “Native Mutators” which evolved with the high mutation from generation 0 (see “Methods”), the deepening of the “U-shape” shows the same symmetric pattern as the controls (Additional file 1: Figure S8). Hence the evolutionary dynamics of the mutator strains is not simply due to the mutational pressure. It is a specific response to the mutational pressure in genomes that evolved without it.

To better understand how this right-skewed “U-shape” evolved in the mutator lineages, we separated the effect of the variation of essential genome length on the local mutational neighborhood from that of the variation of non-essential genome length. To this aim, starting from the 100 mutators lineages at generation 390,000, we randomly reduced the length of the non-essential genome compartment down to its length in the ancestor at generation 300,000 (while leaving essential base pairs untouched and making sure the removal of non-essential base pairs had no effect on fitness – see “Methods”). By comparing this reduced genomes to the ancestors at generation 300,000, we can isolate the effect of the essential genome streamlining on the mutational neighborhood. As expected, the reduction of the essential genome leads to more neutral offspring, hence skewing the “U-shape” left (Fig. 6e, g). Now, by contrasting the reduced genomes to the mutator individuals at generation 390,000, we can isolate the effect of non-essential base pair increase (Fig. 6f, h).

Overall these results show that the increase in non-essential base pairs causes a strong skew that reduces the fraction of neutral offspring (counterbalancing the effect of essential genome streamlining) and increases the highly deleterious ones. When considering only point mutations, this result may seem counter-intuitive. However, in Aevol, organisms undergo different kinds of mutational events, including large-scale rearrangements (duplications, deletions, translocations and inversion). Here the increase fraction of highly deleterious offspring is due to the effect of non-coding inflation on the number and size of these events [22]. Finally, in the bottom part of the “U-shape”, the increase of non-essential base pairs also skews the mutational neighborhood to the right by reducing the number of slightly deleterious offspring and increasing the number of mildly deleterious ones.

### Evolution of fitness at the population level

**Fig. 7:**
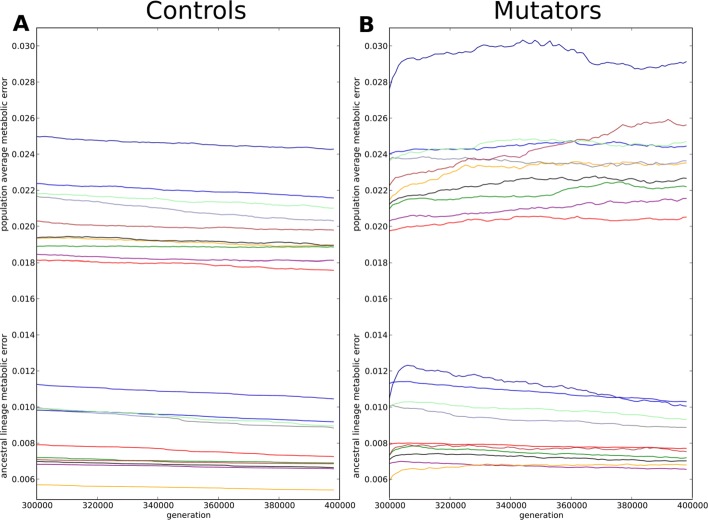
Metabolic error of the populations (top curves) and metabolic error of the ancestral lineages (bottom curves), averaged per WT and per 1000 generations. **a**) Metabolic error of the controls populations and lineages. Both the average metabolic error and the metabolic error of the ancestral lineages decrease in all populations. **b**) Metabolic error of the mutator populations and lineages. While the metabolic error of the ancestral lineage decreases (after the initial increase), the average error of all populations typically increases beyond their initial value at generation 300,000 and goes on increasing even while the metabolic error of the lineage started decreasing: Mutators lineages improve their fitness at the expense of the general population

Throughout the results, we have taken the ancestor as a representative for the population as a whole. Indeed, everything that happens in the line of descent directly affects offspring, and after coalescence, all individuals alive in the population are descendants from the ancestral lineage. But the ancestral lineage does not have to be typical for the population at any given time. Indeed, in our simulations, it appears that the ancestral lineage is not always representative of what happens at the population level: While, in the controls, the fitness of the lineage and the average fitness both decrease in all populations (see Fig. 7a), in the mutators the ancestral lineage improves (hence decreasing its metabolic error) while the average metabolic error of the population rises (see Fig. 7b).

Overall we have seen that, in response to a harsh mutational pressure, mutator populations evolve a new genome structure, hence a new evolutionary dynamic: The coding genome shrinks as coding becomes more efficient, decreasing the risk to be affected by a point mutations. Conversely the non-coding genome increases in size, decreasing the number of neutral offspring and the number of offspring with slightly deleterious mutations and increasing the proportion of highly deleterious offspring. This reduces the average fitness in the population but enables fitness recovery in the ancestral lineage: through evolvability of the genome structure the lineage successfully adapted to the new mutational context.

## Discussion

By evolving virtual bacteria with the mutator phenotype in a constant environment we show that mutator strains initially pay a strong fitness cost, as is classically expected [20]. However, our experiments also show that, after a few thousands generations, all the mutators are able to recover at least partly. More precisely, in 62% of the simulated mutator strains, the mutator were able to recover their ancestral fitness. Moreover, despite mutational load, the lineage of mutators can even reach higher fitness than the wild-type strains that evolve in parallel (25% of the mutator strains). By analyzing the evolution of the mutators genomes during the recovery phase, we have been able to show that in all cases, the mutators adapt to the increased mutation rate by reorganizing their two main genomes compartments: in mean the coding part of the genome (“essential genome”) is reduced by 6% while simultaneously the non-coding part (“non-essential genome”) increased by 86%, resulting in a slight increase of the whole genome (12%).

The model simulates a population of asexual organisms experiencing a strong increase of their point mutation rate. Hence these strains experience the effect of Muller’s ratchet [16]: The strong increased mutation rate leads to the accumulation of deleterious mutations (Additional file 1: Figure S5) and the absence of recombination makes it impossible to eliminate them, hence the initial loss of fitness observed in the populations and in the fixed lineages. Given that the mutation rate is kept high and the environment is constant, the fitness recovery observed in the fixed lineage (but not in the population) is much more surprising. To the best of our knowledge, no previous model ever predicted that mutators could be able to recover their initial fitness when evolving in a constant environment. Classical models predict that mutator strains can become better than wild-types only in case of a sudden change in the position of the fitness peak [28–30]. However these models are generally based on a linear genotype-to-phenotype map that cannot evolve to adapt to the new mutational conditions. This setup enables convenient calculations, but fails to take into account that changing the mutation rate may induce changes on the genotype-to-phenotype map, hence on the fitness landscape, allowing for new discoveries that were previously unavailable.

The model we used, Aevol, is amongst a class of models that have both a complex genotype-phenotype mapping and a complex fitness landscape. This kind of model was first studied in the context of RNA folding [31, 32], but has recently also been applied in studying bacterial evolution [22, 23, 27, 33, 34] (see [35] for a review). In this kind of model, the Genotype-to-Phenotype map offers many degrees of freedom that are accessible to evolution. In particular different sequences can have the same fitness, and many distinct mutants of a same genotype can code for the same phenotype. Even so, these different sequences, although being equivalent in terms of fitness, may differ in their robustness/evolvability properties [22, 36]. In particular, one of the specificities of the Aevol model is to allow for different levels of compactness of its coding sequences: genes can be grouped on operons or overlap, hence increasing the mutational robustness [37, 38], on the opposite, genomes can accumulate non-coding sequences, hence increasing the mutational variability due to large chromosomal rearrangements [22, 26]. Moreover, when compared to simpler models, complex models are likely to show a very different fitness landscape structure [39]. While most models generally suppose a single-peak landscape, complex models are likely to have many plateaus rather than peaks, some being very far away from the others (indeed, when we look at the controls strains, we see that in all cases where populations appear stuck, there is at least one lineage where there is significant improvement, see Additional file 1: Figure S4). We argue that the fitness recovery we observed in our experiments is due to a combination of these two phenomenon: escape of the local fitness plateau to reach another one – higher and/or flatter – through adaptation of the coding structure (reduction and restructuring of the coding compartment), and the gradual increase of the non-coding compartment.

### Mutators adapt their mutational neighborhood

As Fig. 3d and f show, all mutator strains react to the mutator phenotype by (**i.**) decreasing the total number of coding base pairs and (**ii.**) increasing the total number of non-coding base pairs. The former effect is easy to understand through evolution towards robustness [21, 40]: since the number of mutations expected at each replication is proportional to the number of base pairs in the genome and since all mutations affecting *only* non-coding bases are almost surely neutral (the only situation where a mutation in a non-coding base is not neutral is when it creates a new gene, which is highly improbable), the streamlining of the coding sequences offers a protection against the increase of the mutation rate and organisms with less coding sequences are likely to produce more fit offspring. Indeed, such a dynamics has already been observed in digital genetics experiments using the Avida software [41, 42]. Following a similar idea, Marais and Tenaillon have shown, using a error threshold model, that elevated mutation rates can lead to genome streamlining in endosymbiotic and oceanic bacteria [6]. Note however that, given the amount of coding base pairs lost (6% in mean) and the 100 fold increase of point mutation rate, the reduction of the coding sequence is insufficient by far, hence the loss of fitness at the population level (see Fig. 7).

We could not find any positive effect brought by the increase in non-essential base pairs nor any hitch-hiking mechanism that could explain their accumulation. Moreover, as show by Fig. 6f, h, the increase of non-coding sequences is responsible for a reduction of the proportion of neutral offspring (taking up most of the increased neutrality due to the decrease of coding sequences, as seen in Fig. 6e, g), as well as an increase in the proportion of highly deleterious mutants. Despite the fact that mutations exclusively affecting non-coding base pairs do not affect fitness, the number of non-coding base pairs does positively affect the size and frequency of rearrangements per cell, hence indirectly affecting genes [22]. Thus, what could be the benefit of this change in mutator strains? Since the increase of the non-coding genome decreases the fraction of neutral offspring, this is not due to selection for mutational robustness (“survival of the flattest” [21]).

Muller’s ratchet is due to the selection coefficient of the best phenotype being too small to prevent slightly deleterious mutations from being accepted into the lineage. Here, rather than increasing neutrality, the mutator strains evolve a new population structure in which the selection coefficient of the best individual is increased through the reduction of slightly deleterious offspring and an increase of highly deleterious ones. This increases fixation probability of mutants carrying beneficial mutations through the reduction of the mean population fitness. Such an antirobustness strategy, in which individuals are selected for their ability to undergo more heavily deleterious mutations, has been suggested as a mechanism able to mitigate the effect of Muller’s ratchet [43] and has been shown to be selectable for under a soft-selection scheme [44, 45]. Three conditions are necessary for this strategy to be effective: It requires that (1) more offspring are produced than will actually survive at the next generation, (2) survival of a set number of individuals is guaranteed, and (3) competition over the ability to replicate is mostly with closely related individuals. When all these conditions are met, an increase in highly deleterious mutations can be beneficial if the number of slightly deleterious mutations is decreased [45]. In this case, even though the average fitness of the offspring declines, the selective pressure on the fittest offspring increases, which can allow populations to maintain a genome of a larger size than those with a less deleterious local neighborhood.

Our Aevol populations meet all three conditions. At each generation an individual can only replicates on its immediate vicinity, producing a maximum of 9 offspring. However, due to the fixed population size *N*, competition implies that some individual will not replicate at all (condition 1) but that at least *N*/9 individuals will replicate at each generation (condition 2). Finally, both competition and replication are local in the model (hence competition with close kin is common). We also observe the decrease in fitness of the average population while the fitness of the ancestral lineage improves (Fig. 7), as is expected under an antirobustness strategy. This, and the strong skew we observed in the distribution of mutation effects (Fig. 6b, d), strongly suggest that an antirobustness mechanism is at work in our simulations. However, importantly, the fact that mutators benefit from the new distribution of mutation effects does not imply that this distribution is directly selected. Indeed, at least three different mechanisms could account for the increase of the non-coding sequences that leads to the skew, hence triggering the antirobustness mechanism. (1) Antirobust organisms could be directly selected for. However, this is rather unlikely as such a mechanism would be highly sensitive to cheaters (i.e. organisms benefiting from the decrease of the mean fitness without paying the antirobustness cost) (2) The whole population could move to a new peak of the fitness landscape, the shape of which would be more adapted to the new mutation rate (be it lower or higher that the original peak). This mechanism, akin to the “Robustness Drift” proposed by [46] would here be triggered by a change in mutation rate rather than by a change in population size (as in [46]) but the overall mechanism could be similar. Finally, (3) the inflating of the non-coding genome could be due to lower selective pressure. Indeed, in mutator populations, the fraction of neutral offspring is lowered (see Fig. 6a and b), resulting in a smaller effective population size [6]. This is likely to lower the selection for genome reduction, hence leading to inflation of the genome compartments that are not directly affected by the increase of the mutation (i.e. non-coding sequences). Such an effect have been shown to account for genome expansion in subterranean species [47] and preliminary results with Aevol show that the genome structure (including non-coding proportion) is indeed influenced by variations of population size [48]. Note however that, whatever the evolutionary origin of the genome inflation, it results in a reorganization of the genotype-to-phenotype map that ultimately help the mutators overcome Muller’s ratchet.

Unlike previous analyses of antirobustness, our Aevol mutators were able to combine antirobustness with evolution of robustness by evolving their genome structure to affect the distribution of mutational effects. Like the control wild types, mutators deepened the “U-shape” in the frequency of mutational effects, which decreased the fraction of slight to moderately deleterious offspring while increasing the fraction of neutral and highly deleterious offspring. In addition to this, mutators strongly right skewed this “U-shape” further increasing the number of highly deleterious offspring and further decreasing the number of slightly deleterious offspring. This combined shift in mutational effects lead to sustained improving the fitness of the lineage (though not the mean fitness in the population) beyond their initial fitness value. Indeed, when the mutators were able to completely recover, recovery only concerned the ancestral lineage. On the opposite, the average fitness of the population continued to degrade over time (see Fig. 7b). This decrease in the average fitness occurred across almost all mutators, whatever the wild-type. In contrast, the average fitness of the control populations did increase (see Fig. 7a), as they only deepen the “U-shape”. In adapting to the change in mutational frequency, evolving the structure allowed for the very mode of evolution of the populations to be altered.

Finally, to fully understand the dynamics of the mutator strains, we contrasted them against populations that evolved under the high mutational conditions from generation 0 (“Native Mutators”, see “Methods” and Table 2). The evolution of the metabolic error in the ancestral lineage of the Native Mutators shows a dynamic far closer to the controls than to the mutator strains (Additional file 1: Figure S7). Unlike the mutator strains, the gains in fitness made by the “Native Mutator” lineages don’t come at the expense of the average fitness of the populations. That is not to say that they don’t experience the same mutational pressure. As Additional file 1: Table S1 shows it, the coding genome of the native mutators is far more compact than that of either the controls or the mutator strains, and the fittest pre-evolved wild-types were fitter than the“Native Mutators”.

**Table 2:** Per base pair per generation rates of the different types of mutations

Mutation types	Wild types & Controls	Mutators & Native Mutators
point mutations	10^−6^	**10** ^**−4**^
small insertions	10^−6^	10^−6^
small deletions	10^−6^	10^−6^
inversions	10^−5^	10^−5^
translocations	10^−5^	10^−5^
large duplications	10^−5^	10^−5^
large deletions	10^−5^	10^−5^

In terms of genome evolution, the “Native Mutators” behave like the control populations (Additional file 1: Table S2), as they reduce number of non-coding base-pairs. As we might then expect, the evolution of the mutational neighborhood of the “Native Mutators’ matches that of the controls, in that they evolve to symmetrically deepen their “U-shape” (compare Additional file 1: Figure S8 to Fig. 6a, b, c, d). Taken together, this demonstrates that the mutator response to the higher mutation regime is fundamentally different from that of a population that evolved in that regime. Through this new evolutionary dynamic, the mutator strain is able to retain a far larger coding region in the lineage, than would have evolved otherwise, and thereby retain fitness in the lineage despite the raised mutational pressure.

### Limits of the model

As argued above, the main difference between Aevol and “classical” mathematical models generally used to assess the effect of mutator alleles [14, 19, 20] is that Aevol owns many degrees of freedom in the way it encodes its phenotype onto its genotype. This allows, the organisms that evolve in the model to adapt to the new mutation rate by changing their genome structure, hence changing the distribution of offspring fitness. However, this raises the question of whether the dynamics observed in the model would apply in real organisms undergoing high mutation rates.

First, as for all models, one could of course argue that the model has a limited degree of realism. In particular, one could argue that the population size (10^3^ individuals) is much smaller than the LTEE population (of the order of 10^7^ individuals) and that there is not enough degrees of freedom in Aevol – or not the correct ones. In effect, though more complex than mathematical models, Aevol organisms are still far from the complexity of real bacteria (e.g. there is no regulation networks nor metabolic network in our model). Indeed, a real organism could escape the elevated mutational load by adapting its genotype-to-phenotype map at other levels than the decrease/increase of coding/non-coding genomic compartments or in different respective proportions, which would result in different trends for genome size. In the LTEE, for instance, mutator strains could have evolved robustness by contracting their genome (as shown in [49]) and antirobustness strategies by acting at other levels (e.g., reorganization of their regulation network [50, 51]). Moreover, in our model the increase of the mutation rate comes with no bias while “real mutators” show strong biases [52], including elevated rate of intra-chromosomal recombination [53]. Such biases are likely to limit genome expansion as an elevated rate of rearrangements imposes strong robustness constraints on the overall genome length [22]. Finally, our simulations differ from the LTEE on an important point: In our simulations, the mutator identity is acquired while the organisms evolve in a constant environment (contrary to the LTEE in which the experiment started with an environmental change even though the environment is fixed thereafter). Hence beneficial mutations are less likely. Yet, still the dynamics observed here is of great interest since it shows that the deleterious effects of an increase of mutations on the lineage can be mitigated by changing the shape of the distribution of offspring fitnesses (the “U-shape”), whatever the (molecular) mechanism is. Indeed, such an effect has been observed is other situations and in other models [27, 54], showing that this distribution is a target of second-order selection and that it’s shape could evolve as a reaction to environmental or mutational stress.

Second, it must be acknowledged that the situation modeled in our experiments is somewhat artificial. Indeed, in our experiments all the population acquires the mutator allele at once and there is no competition between mutators and non-mutators (in that sense the situation is similar to the LTEE *after* mutators have established). Moreover, individuals cannot lose their mutator identity by e.g. breaking the linkage between the mutator allele and the beneficial mutations [55] or by evolving compensatory mutations [12]. However, the interest of our experiments is precisely to show what could be the fate of mutators *if* they succeed to maintain long enough in the population. This can only be studied through the kind of “impossible experiments” we performed here: by modeling an artificial situation, we have been able to show that the mutational burden is escapable, even in the absence of recombination, and that bacteria evolving under a very high mutational stress can thrive by rearranging their genotype-to-phenotype map (here their genome architecture). In other words, organisms are able not only to adapt their mutation rate to the environmental conditions, they are also able to adapt *to* the mutation rate, a hallmark of the “Evolution of Evolution” (aka “EvoEvo”) process where evolution is itself changed by evolution [35]. Doing so mutators are able to take the better of the two states: increased probability to find improvements and reduced deleterious mutational load.

## Conclusion

By evolving in silico populations of mutators in a constant environment, we have observed a striking dynamics with the ancestral lineage of mutators experiencing a fitness loss lasting a few thousands of generations but then being able to recover and, in a vast majority of the lineages evolve back to their initial fitness. By analyzing the dynamics of their genome along the lineage, we have shown that this dynamics is due to a combination of three processes: (**i.**) Mutation accumulation that accounts for the fitness loss since our populations experience Muller’s ratchet, (**ii.**) coding sequences compaction that reduces the genomic target to mutations, hence reducing the mutational load, and (**iii.**) non-coding sequences inflation that skews the distribution of offspring’s fitness (the “U-shape”) and increases the selection coefficient. Taken all together, these three processes show that, even though a clonal population acquiring the mutator phenotype is likely to pay a strong initial fitness cost, this cost can be escaped. It may be compensated by a reorganization of the individual’s genotype-to-phenotype map that can lower the effect of the mutations (evolution towards robustness), increase their effect (antirobustness) or *even* a combination of the two as we observed here. In our model, the main degrees of freedom for the genotype-to-phenotype map is the genome structure and we indeed observe changes at that level. In real organisms the genotype-to-phenotype map contains many different levels (genome, regulation network, metabolic network...) that can all be reorganized in order to increase or decrease the effect of mutations. Hence, depending on the organisms and/or the environmental conditions, the adaptation of the genotype-to-phenotype map could take place at any of these levels and not, or not only, at the genomic level as observed here. Nevertheless, the simple fact that a reorganization of the genotype-to-phenotype map can counterbalance the mutational load is a important discovery that is likely to change our view on mutator strains: contrary to what is classically expected, mutators could very well last in population long after an environmental change has occurred by structurally adapting to their high mutation rate. Moreover, our results emphasize the importance of evolution of genotype-to-phenotype map and its contribution to the evolutionary dynamics.

## Methods

### Aevol modeling formalism

**Fig. 8:**
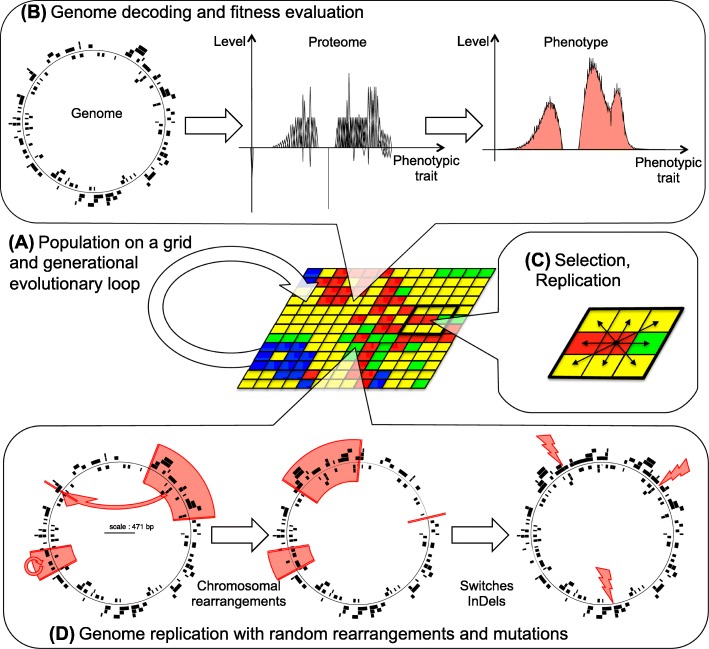
Overview of the Aevol model (**a**) The population is contained on a grid. It reproduces at each generation with complete replacement. (**b**) Each individual owns a circular genome with scattered genes identified through initiation/termination signals (promoters, terminators, RBS, Start/Stop codons). Genes are translated into a set of proteins, each one contributing to some phenotypic traits with a certain level (phenotypic trait, level of contribution and level of pleiotropy are encoded in the gene’s sequence). All proteins are grouped to form the phenotype and the difference between the phenotype and the environmental function (light red) gives the metabolic error (see main text for details). (**c**) Each individual competes with its local Moore neighborhood. An individual can have 0 (if it looses all the replication competitions it participates to) to 9 (if it wins all the replication competitions) offspring at each time steps. (**d**) During replication, the genome can undergo large chromosomal rearrangements (here an inversion and a translocation) and small mutations (switches, InDels)

Aevol is an individual based model, where population of virtual organims are submitted to variation and selection processes (Fig. 8). The specificity of Aevol is that organisms own a genome that structurally mimics bacterial genome organization (Fig. 8b) and that this genome is altered by variation operators that also mimics bacterial ones (Fig. 8d). Hence, it is possible to study how genome organization impacts evolution and, in turn, how evolution influences genome organization. In Aevol each individual contains a circular double-strands genome composed of a binary bases. To calculate the cells properties, this genome is read for promoters (promoter being 22 bp consensus sequences, the Hamming distance to the consensus driving the transcription rate of the promoter). The sequence following the promoter is transcribed until a terminator is reached (a terminator being a sequence able to form a stem-loop structure analog to *ρ*-independent terminators in bacteria). If the transcribed RNA contains a Ribozome Binding Site (RBS) followed by a Start codon, translation will start until a Stop codon is reached on the same reading frame. An RNA can contain multiple genes and a gene can be contained in multiple RNA’s depending on the distribution of promoter and translation initiation sequences.

**Fig. 9:**
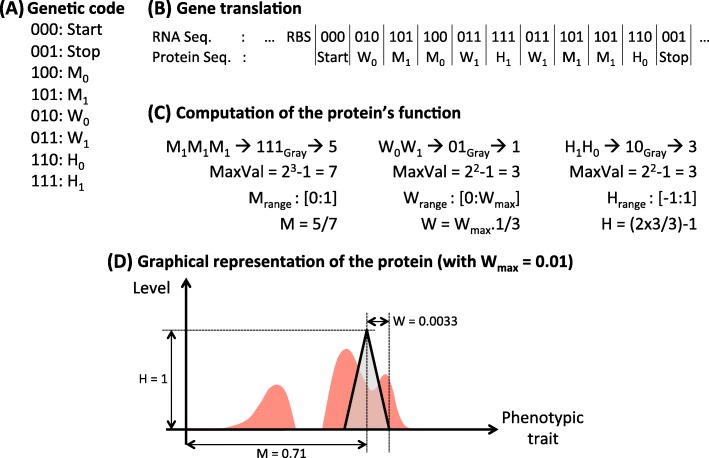
The translation process in Aevol. **a** Aevol’s genetic code. The code contains 2^3^=8 non-redundant codons, two of which encoding for START and STOP (000 and 001 respectively). The six remaining codons encode for six “Amino-Acids” (AA) that are pairwise associated to three classes respectively coding for *M* (AA *M*_0_ and *M*_1_), *W* (AA *W*_0_ and *W*_1_) and *H* (AA *H*_0_ and *H*_1_). **b** Translation of the gene into the protein’s primary structure. The transcribed RNA sequence is parsed for Ribosome Binding Sites (RBS) followed by a START codon. The translation then started on the reading frame of the START codon until a STOP codon is found (note that the length of the gene is not predefined). The protein’s primary structure is a sequence of AA whose length depends on the gene’s length. **c** Computation of the protein’s parameters. The AA sequence is parsed into three substructures containing respectively all AA of class “M”, “W” and “H”. These substructures are then translated into binary sequences that are converted into an integer value via Grey code. This value is turned into a fraction that determines the actual value extracted from the range of possible values of that property. **d** Graphical representation of the protein function. The protein is represented as a triangle defined by three properties: Main phenotypic trait (*M*), level of contribution to the trait (*H*) and level of pleiotropy (*W*). For sake of clarity, the phenotypic target is also represented on the figure (see Fig. 8). Note that the *H* value may be scaled by the activity of the gene’s promoter

**Protein properties.** Genes are made up of three base long codons (hence, with a binary genome, there are 2^3^=8 different codons, see Fig. 9a). Translation starts at the START codon and then continues until the STOP codon is encountered on the same reading frame, producing a protein which primary structure is a chain of “Amino-Acids” (with an alphabet composed of six AA, see Fig. 9a and b). In Aevol, we define a mathematical space in which all possible phenotypic traits are expressed by real values in [0,1]. Then a protein can be represented by a [0,1]→[−1,1] function representing the distribution of phenotypic traits it contributes to and their level of activation (negative values representing inactivation of the corresponding traits). In Aevol this distribution is triangular, hence defined by three parameters: its position on the phenotypic trait axis (main trait the protein contributes to), its height (level of contribution of the protein to the trait) and its width (level of pleiotropy of the protein). For each property, two codons translate into amino acids which affect that property based on their order in the gene (see Fig. 9a and c). Each property has its own range of possible values. To find out what value the series of amino acids codes for, we convert the series of binary values by Gray code (Fig. 9c). The resulting integer is then normalized and the result defines the value of the corresponding protein/triangle parameter (Fig. 9d).

**Phenotype** The phenotype of an individual is the sum of all the traits its proteins activate (1 being the maximum activation value) minus the sum of all the traits its proteins inhibit (traits that are more inactivated than activated being set to 0). The phenotype is thus a [0,1]→[0,1] multilinear function.

**Environment and selection.** In Aevol the environment in which the organisms live is indirectly represented by the optimal [0,1]→[0,1] phenotypic function its allows. In all experiments presented here we used a constant target function (in light red on Figs. 8b and 9d) which is the sum of three Gaussians (see Table 3), two positive and one negative.

**Table 3:** The properties of the three Gaussians whose sum makes up the fixed environment

Gaussian ID	top height	Mean	Standard deviation
1	1.2	0.5	0.12
2	-1.4	0.52	0.07
3	0.3	0.8	0.03

The phenotype of an organism and the phenotypic target are compared to compute the organism’s “Metabolic Error” (i.e. the integral of the difference between both functions). This metabolic error is then converted into an exponential fitness value. Note that the metabolic error *decreases* when the individual becomes fitter.

The entire population is placed on a toroidal grid with one individual per grid cell. The size of the grid thus specifies the size of the population (in all experiments presented here we used a 32×32 grid, i.e. 1024 individuals). Aevol uses a generational algorithm: at each generation each individual competes with its local Moore neighborhood (Fig. 8c). The probability of reproduction is proportional to the fitness (i.e. inversely proportional to the metabolic error) and a biased random wheel is used to select for the individual that will occupy the grid cell at the next generation.

**Mutations** During replication, individuals’ genomes may undergo variation through mutations. Seven types of mutations have been used in our simulations, three being small scale mutations and four being large scale mutations. The small scale mutations are: point mutations, small insertions and small deletions (InDels). Point mutations switch a single base pair into the opposite base (on both strands), while small duplications and deletions respectively insert or remove 1 to 6 bases at a random location. Large scale mutations consist of large duplications and deletions, which duplicate or delete a section of the genome between two uniformly drawn breaking points, and translocation and inversion mutations which cut out a section of the genome and insert it somewhere else or inverts the two strains respectively (see Fig. 8d). All mutation rates are specified independently and the rates are defined per base-pair per generation.

### Experimental setup

We used the in silico Experimental Evolution strategy described in [23]. Wild-type organisms are evolved in Aevol under constant conditions for a very long time starting from 5000 bp long random sequences having at least one gene (naive individuals). Then the Wild-Type populations are cloned and evolution is resumed under different conditions to isolate the effect of the conditions on evolution pace or on the structure of the organisms. Here, we evolved 10 wild-type clones for 300,000 generations in a single stable environment, with a population size of 1024 individuals (32 ×32 grid) and a mild mutation rate (10^−6^ mutations per bp per generation for all local mutations and 10^−5^ mutations per bp per generation for all large scale rearrangements). At generation 300,000 the 10 populations have been replicated 20 times each. Half of these replicates have had their point mutation rate increased 100 fold (up to 10^−4^ mutations per bp per generation, all other mutations rates being kept constant) and let evolve for 100,000 generations in the same environment (these replicates are later-on referred as “mutator populations”). The other half of the replicates continued their evolution for 100,000 generations under the same mutational pressure as the Wild-Types and in the same environment (“control populations”). All mutation rates used in the experiment can be found in Table 2. This experimental setup is similar to the one used in [56] and enables us to study the effect of prolonged hyper mutator identity on initially well adapted populations.

Additionally, we evolved ten wild-type populations under mutator conditions for 400,000 generations in a constant environment (“native mutators”). These populations were used to compare the fate of newly evolved mutators with organisms that constantly evolved under a high mutational pressure.

**Reconstruction of ancestral lineages and ancestral clones.** To analyze the evolutionary fate of the different clones, we reconstructed ancestral lineages from the best individual at the end of generation 400,000 to the beginning of the clones evolution at generation 300,000. Ancestral lineages were reconstructed for the mutator and control populations. Once the ancestral lineages have been recovered, we are able to reconstruct all the mutational events that led to the best final organism in each clone. We then systematically excluded the last 10,000 generations (generations 390,000 to 400,000) to ensure that all observed clones and mutational events have come to fixation.

**Quantification of essential and non-essential genomic sequences.** In Aevol the genome is composed of several structures that mimics real genomic compartments in prokaryotic organisms. In particular, one can distinguish the transcribed sequences from the non-transcribed ones and the translated sequences (ORF) from the non-translated ones. We used two aggregated measures, namely the essential and non-essential genome length. Essential genome contains all sequences that contribute to the phenotype of the organism (i.e. a mutation in essential genome will generally modify the phenotype). Essential genome thus contains promoters and terminators of coding RNAs as well as the Ribosome Binding Site and the ORF for all genes. Non-essential genome contains the rest of the sequence (i.e., non-transcribed sequences, non-coding RNAs and the leaders and trailers of coding RNAs). A mutation in non-essential genome is very likely to be neutral (it can be non-neutral only if it spontaneously creates a new gene).

**Genetic engineering of the clones.** In order to study the consequences of the accumulation of non-essential sequences, we developed a tool that reduces the length of the non-essential genome down to a predefined value without modifying the essential part of the genome. From the evolved strain, we randomly remove one base-pair from the genome and compute the metabolic error of the resulting strain. If the metabolic error is different than the original one, the base-pair is reintroduced in the genome and another one is tested. If the metabolic error is conserved, then the base-pair is definitively removed from the genome and we start searching for another one to remove. This procedure is continued until the length of the non-essential genome reaches the desired value (typically the length of non-essential genome in the ancestor of the strain).

**Quantification of the mutational neighborhood.** Given the complexity of the genotype-to-phenotype mapping, the combinatorics of the genomic sequences and the diversity of the mutation operators, it is impossible to characterize the whole fitness landscape of Aevol’s organisms (exactly as for real organisms). Therefore we developed a procedure that sample the local structure of the fitness landscape around a given position. To this end, given an individuals genotype, we used the same replication procedure as the one used during the evolutionary experiments (including the same mutation procedure) and produced large number offspring of this individual (2 million in the current experiment). We then measured the fitness distribution of these offspring. This distribution represents the local structure of the fitness landscape (note that this structure is dependent on the mutation rates). By comparing the neighborhoods of different individuals, we can gain an understanding of the effect of changes that don’t directly affect fitness but that nevertheless modify the effect of mutations (e.g. evolution of genome structure).

## Supplementary information

**Additional file 1** Supplementary file 1.

## Data Availability

The datasets used and/or analysed during the current study are available from the corresponding author on reasonable request. The aevol program and the data generation tools are available at http://aevol.fr/publications/resources/aevol_rutten_et_al.zip

## Abbreviations

LTEE: Long term evolutionary experiment
