# Supplementary material for: Adapting the engine to the fuel: mutator populations can reduce the mutational load by reorganizing their genome structure
Source: BMC Evol Biol. 2019 Oct 18;19:191. doi: 10.1186/s12862-019-1507-z (PMC6800497; doi:10.1186/s12862-019-1507-z)
Supplement: Supplementary file 1 — Additional file 1 Supplementary file 1. [file 12862_2019_1507_MOESM1_ESM.pdf]

Supplementary file 1

Supplementary Figure 1

Evolution of the wild-types occurred in two phases. An initial phase of rapid increase in fitness and phase of increasingly punctuated equillibria separated by moments of fitness increase. Unlike the analyses in the rest of the paper, here we are plotting the best individual of each population.

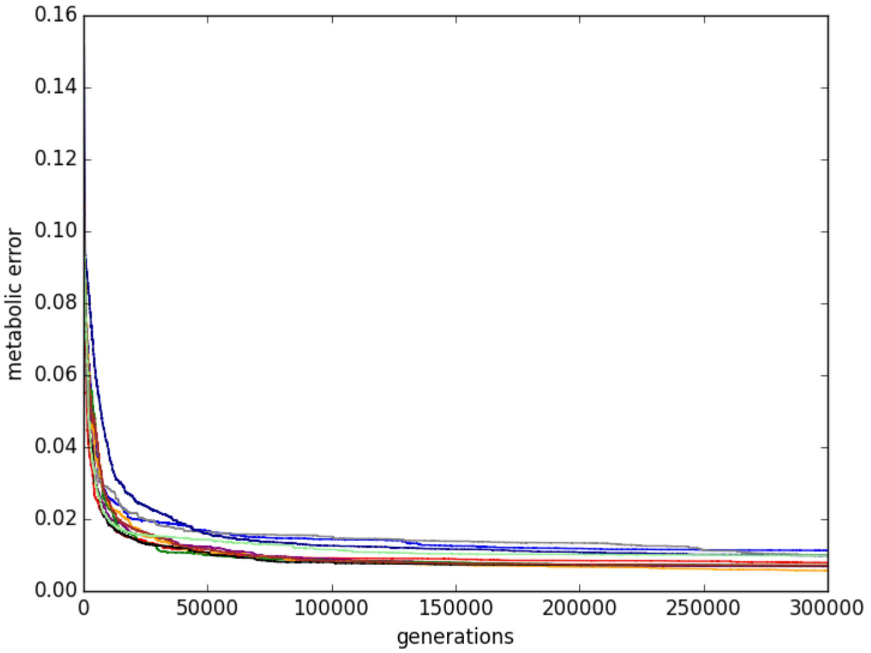

Supplementary Figure 1 Evolution of the metabolic error of the best individual for each Wild-Type. Most fitness gain is made during the first 100,000. After this first period of fast fitness improvement, evolution slows down and very few progress is made during the last period, showing that the WT strains are almost stabilized at generation 300,000.

Supplementary Figure 2

The mutator lineages all underwent an initial period of fitness loss before they started to regain fitness. 77% of the strains have started their recovery by generation 30,000, but in the fittest wild-type 5 (yellow) even the fastest recovery happens after 30,000 generations, leaving them with less time to recover.

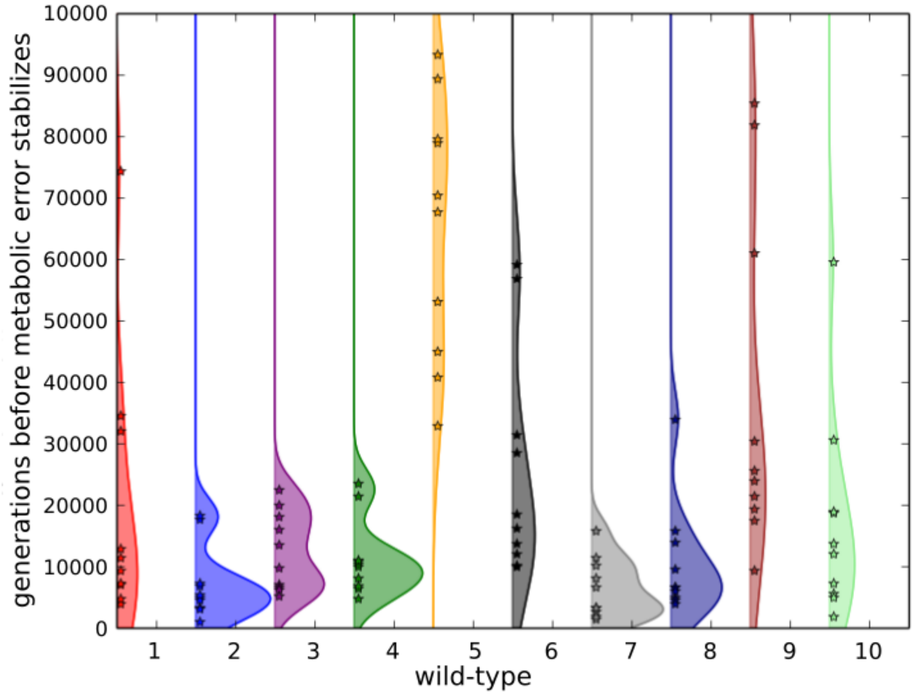

Supplementary Figure 2 Distribution of the time it takes before the metabolic error of the ancestral lineage starts to improve for each mutator population. The stars represent the replicates in each population. Most of the ancestral lineages have started to improve after 40,000 generations, but population 5, which is the fittest wild-type, generally takes far longer, leaving less time for the recovery.

Supplementary Figure 3

The mutators gained base pairs gradually across all wild-types. There are multiple mutational sources to gain or lose base pairs, particularly non-essential base pairs. Here we show that there is a clear positive bias in the cumulative effect for large dups/dels. The hypothesis that mutator strains are growing because they are unable to completely repress duplications from adding base pairs is refuted by the fact that the cumulative effect of small dups/dels remains around zero, when we would expect them to clearly bias towards deletion. In combination with figure **??**, which showed that the direct effect of duplications to only rarely be directly positive, this instead suggests an increase in base pairs is selected for.

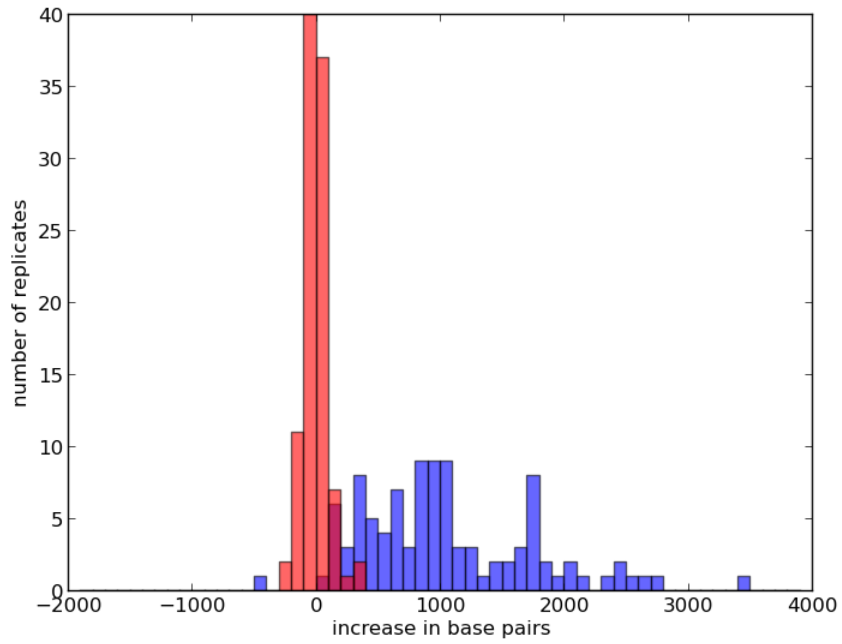

Supplementary Figure 3 Distribution of the total effect on the base pairs in the ancestral lineage of replicates. The effects of small dups/dels is given in red, and the effects of big dups/dels are given in blue. The large increase in non-essential base pairs observed in mutators is due to the increase

Supplementary Figure 4

One hypothesis for why the ancestral lineage of mutators is able to become more fit than that of the controls (see figure **??**) is that the controls could be stuck on a local optimum, while the mutator is able to reach or stumble down and walk back up beyond a fitness valley out of reach for the control strains. Here we show that amongst the control strains, each wild-type contains at least one strain whose metabolic error is clearly reduced, demonstrating that none of the wild-types were at a true local optimum.

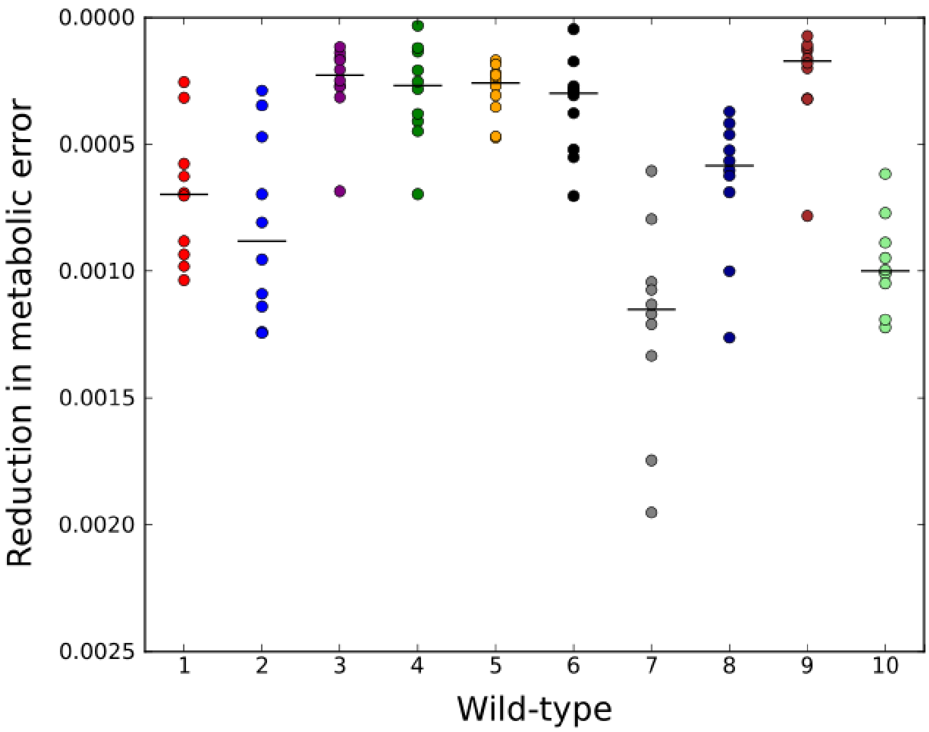

Supplementary Figure 4 Reduction of metabolic error for each of the wild-type control. Even amongst the wild-types that generally do not improve in fitness, there is at least one population that did still improve considerably, demonstrating that these populations are not truly stuck on the local optima.

Supplementary Figure 5

All mutator lose essential basepairs during their first 10,000 generations, at which point the number has stabilized for most wild-types. To see how this affected the accumulation of point mutations, the most common mutation type for mutator strains, we plotted the net effect of point mutations accepted into the lineage over 10,000 generation timespans. We see that in the initial 10,000 generations the mutational pressure forces negative mutations into the lineage, while after this the wild-types tend towards reducing their metabolic error.

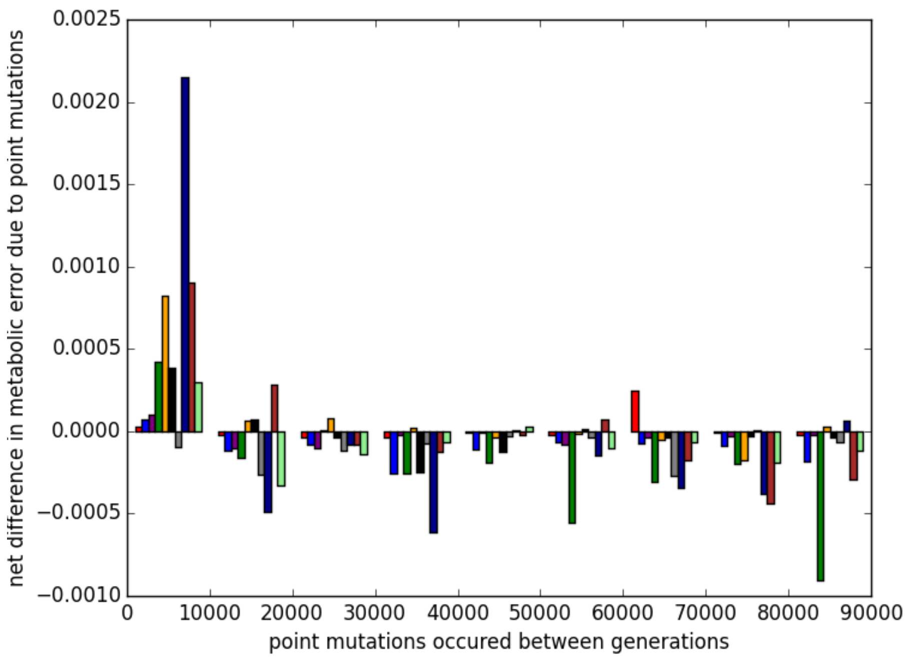

Supplementary Figure 5 Cumulative effect of point mutations accumulated in the ancestral lineage per 10,000 generations in the Mutator strains, averaged per wild-type(colored and ordered as in previous figures) . In the first 10,000 generations point mutations the net effect of point mutations is an increase in the metabolic error in all populations (except for 7, which has already recovered its shallow increase by generation 10,000). In the following time intervals we see that the accepted mutations tend to be neutral to slightly negative, demonstrating that the ancestral lineage is able to maintain itself from this point onwards.

Supplementary Figure 6

Individual mutator and control metabolic error in the ancestral lineages. In the mutator strains we see that all strains struggle to retain their fitness as the metabolic error climbs and falls relatively gradually across all strains. In the control strains on the other hand, the metabolic error either remains stagnant or decreases in a stair like fashion.

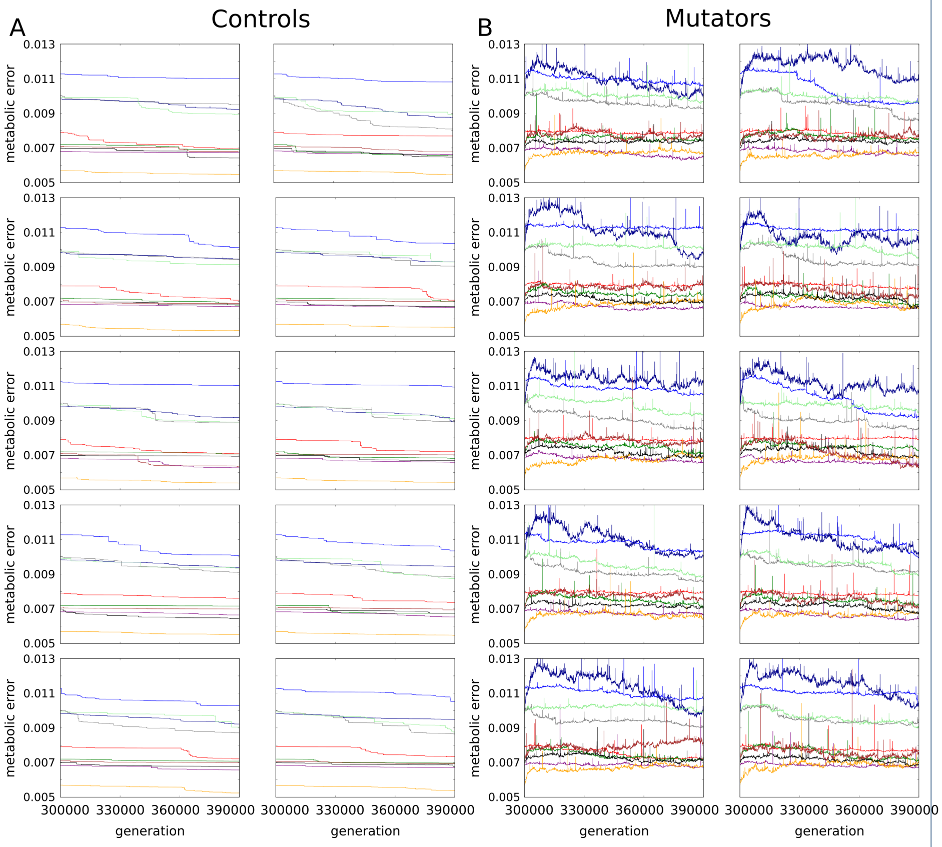

Supplementary Figure 6 Metabolic error in the ancestral lineage per duplicate for the controls(A) and the mutators(B). In the controls we see that the metabolic error remains completely static between the generations where a reduction takes place. In the mutators not only do deleterious mutations appear in the lineage from generation to generation (sudden spikes), there are also longer periods of fitness loss even after the population starts to recover.

Supplementary Figure 7

To better understand the behavior of the mutator strains, we also grew 10 wild-types in the hyper mutation regime from the start of evolution. Like the mutator strains, the ancestral lineage of native mutator wild-types sometimes includes reductions in fitness, but by and large native mutators are far more capable of maintaining their fitness level than the mutator strains. Additionally, while the average population fitness drops in the mutator strains(see **??**B), the average population fitness slightly increases in most native mutator wild-types. This suggests that the evolutionary dynamics of the native mutators are closer to that of the controls than that of the mutators whose mutational dynamics they share.

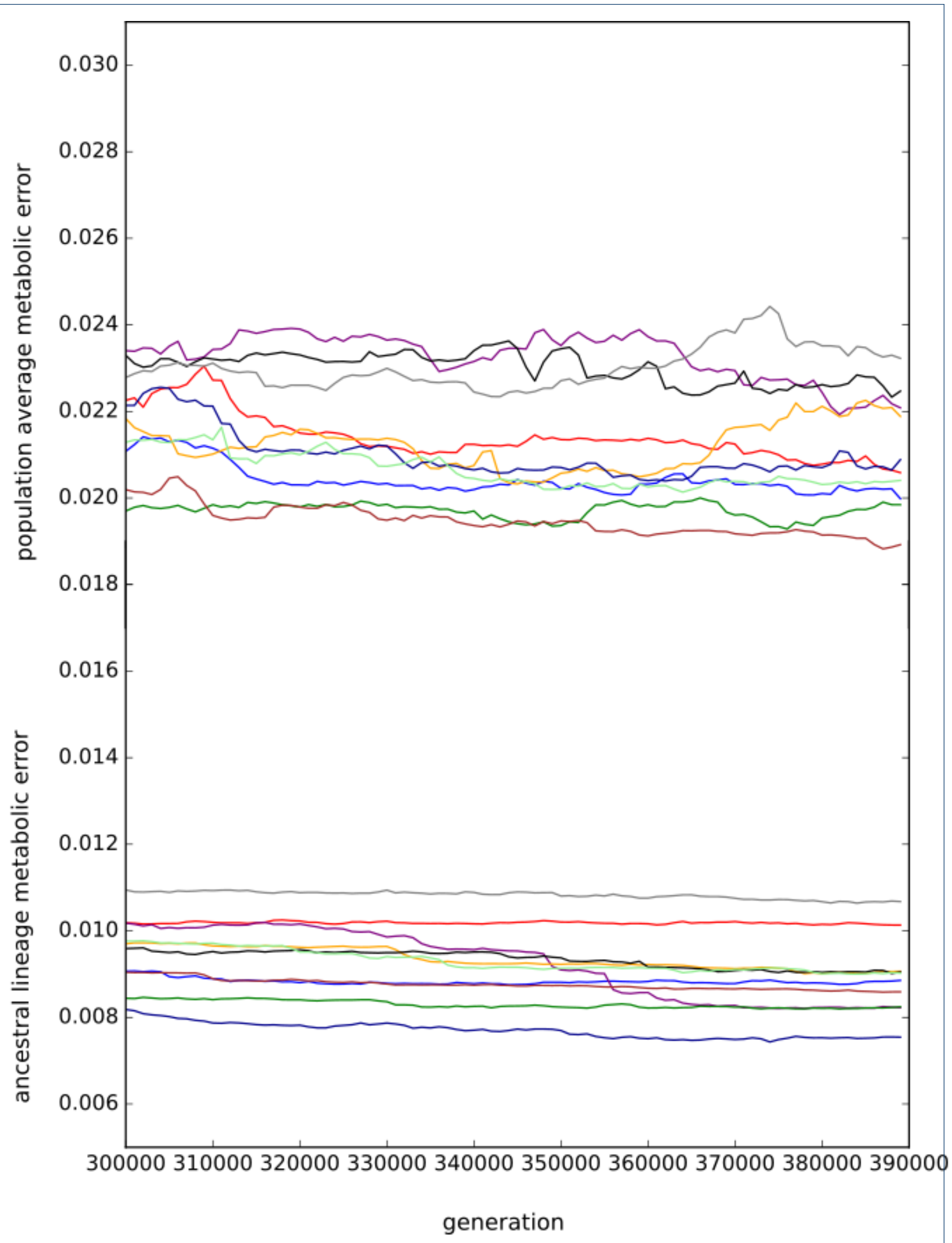

Supplementary Figure 7 The average metabolic error of the native mutator populations plotted in the same figure as the metabolic error of the ancestral lineages, averaged per 1000 generations. The metabolic error of the mutators is lower than that of the worst adapted controls, but higher than that of the best adapted control populations. Like the controls populations the native mutators gradually improve over time, retaining their error level between improvements. While the average metabobolic error is more erratic (these are the averages of a single population, not that of 10 duplicates as in the control and mutator experiments), most of the populations that improve in the ancestral lineage (black, purple,darkblue, brown, lightgreen) also reduce their metabolic error in the average population and there is no clear upwards trend like displayed by the mutator populations in any of the native mutators populations. Despite sharing the same mutations as the mutators, the evolution of native mutators appear more similar to the controls.

Supplementary Table 1

Comparing the native mutators to the mutator and control strains we see that the native mutators are far more compact, particularly in the number of essential base

pairs. Additionally, the increase in non-essential base pairs has driven the coding fraction of the mutators to below that of the native mutators, whose number of non essential base pairs are far closer to those of the controls.

Supplementary Table 1 Number of base pairs, split up between essential and non essential and their respective fractions. The native mutators have fewer essential base pairs than the control and mutator clones. The number of non essential basepairs in the native mutators lies closer to that of the controls than that of the mutators, and correspondingly its essential fraction is highter than that of the mutator clones, despite being lower than that of the controls, which streamline their genome more effectively.

background	total bp	essential bp	non essential bp	essential fraction
controls	7877.95	6547.65	1330.30	0.83
mutators	9005.87	6166.02	2839.85	0.69
native mutators	6458.00	4756.40	1701.60	0.74

Supplementary Table 2

In terms of changes in the ancestral genome over the last 90,000 generations, the native mutators have grown closer to the control strains, while the mutators have evolved in the complete opposite direction. Unlike both the control strains with whom they share a long evolutionary history, and the native mutator with whom they share their mutational regime, the mutator strains are clearly decreasing their number of essential base pairs and rapidly increasing the number of non essential base pairs.

Supplementary Table 2 Average changes in then number of base pairs for the controls, mutators and the native mutators. These differences are further split up between essential and non essential. The standard deviation is given in brackets. Both the control and native population tend to decrease their non essential base pairs, streamlining their genomes, as their essential genome remains relatively stable. In the mutator strains on the other hand we see a clear decrease in the number of essential base pairs, while the number of non coding base pairs rises rapidly.

background	total bp		essential bp		non essential bp	
controls	-171.85	(178.50)	31.49	(73.62)	-203.34	(139.40)
mutators	954.37	(515.84)	-359.59	(321.52)	1313.96	(645.83)
native mutators	-402.30	(491.42)	-9	(51.75)	-393.3	(511.44)

Supplementary Table 3

Inspecting the coding structure on a closer level we see that the mutator strains are far more similar to the controls than to the native mutator, both at the RNA and the gene level. Native mutators mainly have fewer genes and RNAs than the control and mutator strains.

Supplementary Table 3 Markers for coding structure at generation 390,000 for the controls, the mutators and the native mutators. Between the controls and the mutators, the difference in the number of genes and RNA is larger than the difference between the number of base pairs per gene and RNA. After 90,000 generations under mutator conditions, the coding structure of the mutators is still closer to that of the controls than that of the initial mutators in all categories.

Background	Gene number	bp per gene	RNA number	bp per RNA	Genes per RNA
controls	98.55	61.00	50.11	256.26	1.97
mutators	92.01	59.52	47.25	253.16	1.95
native mutators	70.70	57.64	42	227.76	1.72

Supplementary Table 4

We contrasted the average number of mutations accepted into the lineage per mutation type for the controls, mutators and native mutators respectively. As expected, The effect on the point mutations is the largest, with both the mutators and the native mutators having over 100 times more accepted point mutations than the controls. The increase of accepted mutations for all other mutation types in mutators and native mutators is due to lower selectivity. We can also see that the bias towards large duplications over large deletions is present only in the mutators, and not in the native mutators.

Supplementary Table 4 Average number of mutations acquired per mutation type in the final 90,000 generations in the lineages of the controls, the mutators and the native mutators respectively.

			Controls			
population	point	indel	large dup	large del	translocation	inversion
controls	160.9	287.2	7	10.1	2.9	110.6
mutators	28333.4	513.4	34.3	25.6	41	468.3
native mutators	19901.6	382.8	26.9	25.8	32.6	335

Supplementary Figure 8

A more detailed look into the mutational dynamics of the native mutators demonstrates that they have a classic “U-shape”, where the neutral and highly deleterious offspring increase as the number of intermediately deleterious mutations is reduced.

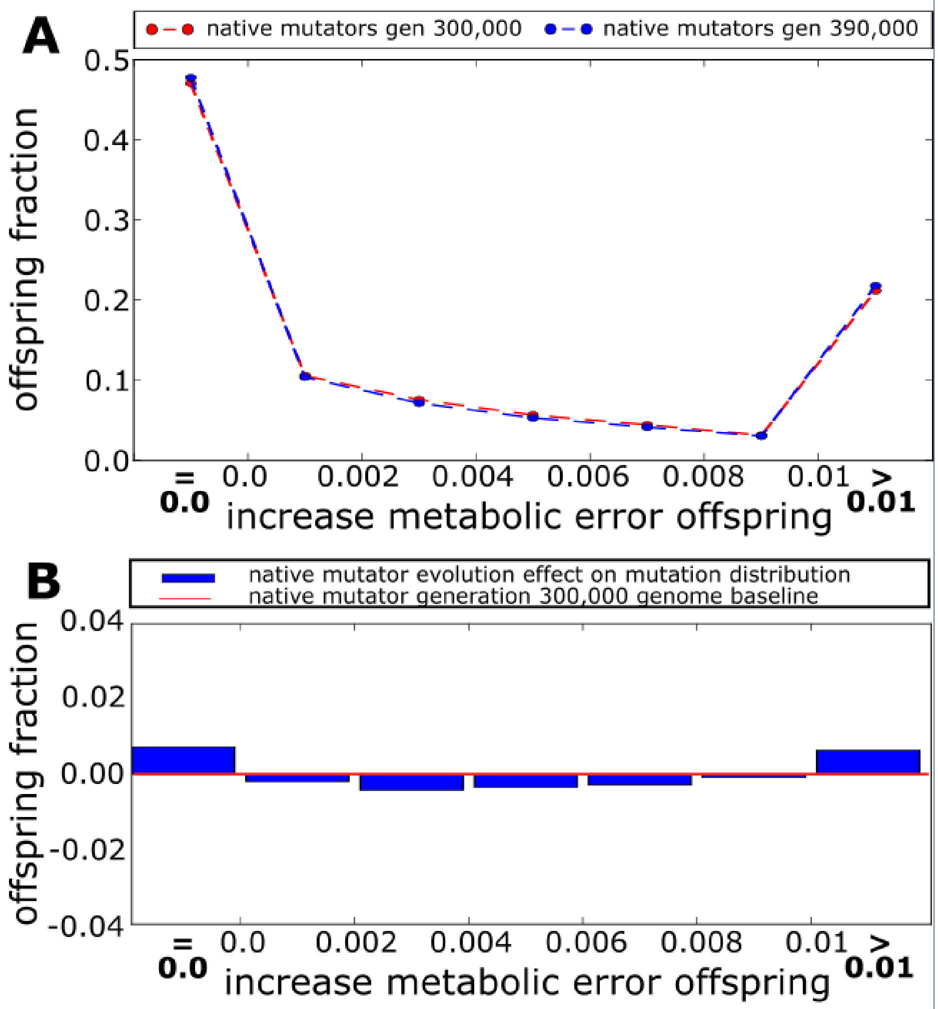

Supplementary Figure 8 Evolution of the mutational neighborhood of the native mutators. (A) Between generation 300,000 (red) and generation 390,000 (blue) evolution deepens the “U-shape” in the distribution of offspring symmetrically (B). Both distributions are binned in 7 bins. The first bin contains neutral offspring ($\Delta g = 0$). Bins 2 through 6 contain the intermediately deleterious offspring binned in 0.002 intervals (Δg from 0.002 to 0.004; 0.004 to 0.006; 0.006 to 0.008 and 0.008 to 0.01). The last bin contains all heavily deleterious offspring ($\Delta g > 0.01$).
